# Rehospitalization and “Revolving Door” in Anorexia Nervosa: Are There Any Predictors of Time to Readmission?

**DOI:** 10.3389/fpsyt.2021.694223

**Published:** 2021-07-23

**Authors:** Enrica Marzola, Paola Longo, Federica Sardella, Nadia Delsedime, Giovanni Abbate-Daga

**Affiliations:** Department of Neuroscience “Rita Levi Montalcini”, Eating Disorders Center, University of Turin, Turin, Italy

**Keywords:** eating disorders, drive for thinness, treatment, readmission, body image, depression, anxiety, body dissatisfaction

## Abstract

**Objective:** Anorexia nervosa (AN) is a severe psychiatric illness with multifactorial etiology and unsatisfactory treatment outcomes. Hospitalization is required for a substantial number of patients, and readmission (RA) commonly occurs. Some individuals need multiple hospitalizations sometimes over a short amount of time, thus, delineating the “revolving door” (RD) phenomenon. However, very little is known about readmissions and their frequency in AN. Therefore, we aimed to longitudinally investigate readmissions in AN in order to: (a) characterize patients with AN who need readmission (i.e., RA-AN), sometimes rapidly (RD-AN); (b) ascertain differences between RA-AN and non-RA-AN groups during baseline hospitalization; (c) investigate as to whether clinical or psychometric parameters worsened on RA; and (d) analyze predictors of time-to-readmission in AN.

**Methods:** A total of 170 inpatients with AN were enrolled at their baseline hospitalization; all their subsequent rehospitalizations were recorded with a longitudinal design by which each patient has been observed for 3 years. Patients were classified as RD-AN if requiring a readmission <12 months since last discharge. Clinical characteristics were measured upon admission and discharge for each hospitalization, and at all time points, patients completed questionnaires assessing eating and general psychopathology, and body shape concerns.

**Results:** Sixty-seven patients (39.4%) needed at least one readmission and 62 (92.5% of RA-AN) reported RD. Compared with non-RA-AN, those with RA-AN were younger, reported a shorter duration of illness, and were more frequently diagnosed with AN-BP. Also, greater severity of anxious and depressive symptoms and body shape concerns emerged in the RA-AN group. The outcome of baseline hospitalization did not differ between groups, and only depressive symptoms worsened at readmission. Shorter duration of AN and low weight gain during baseline hospitalization predicted early readmission but did not survive statistical control. In contrast, high scores on drive for thinness upon baseline hospital entry robustly predicted a shorter time to readmission even after statistical control.

**Discussion:** Individuals with AN who require readmission do so over a short period notwithstanding a positive treatment outcome during their baseline hospitalization. Shorter time-to-readmission can be predicted mostly in case of marked drive for thinness and poor weight gain at baseline hospital admission.

## Introduction

Anorexia nervosa (AN) is a severe mental disorder with multifactorial etiology, highly peculiar patterns of eating behaviors, and psychiatric and organic comorbidities. Mortality is high, also as a consequence of organic conditions sometimes coupled with suicidal ideation (1), mostly for those patients severe to the point of requiring hospitalization (2). Currently, treatments are poorly satisfactory (3), with long-term recovery outcomes of ~60% (4). Despite a wide agreement on the need for treating patients as much as possible in the outpatient setting, not only because of cost effectiveness but also to minimize treatment-related social isolation, in an increasing number of cases, hospitalization is required, even for the youngest patients (5).

Per international guidelines (6), hospitalization in AN should be considered to provide medical stabilization and initiate refeeding, when the physical health of the patients is compromised to the point that their clinical management becomes unbearable in the outpatient setting. Moreover, an absolute weight or body mass index (BMI) threshold should not be used to require hospitalization since the rate of weight loss could be much more relevant than BMI itself (6). In addition, the exacerbation of AN could put patients at risk of suicidal crisis that hospitalization could mitigate instead. Still, hospitalization becomes an option for all patients who experience high difficulties with treatment adherence (i.e., following eating plans while at home) because of partial motivation or environmental obstacles leading to psychic and physical consequences. Therefore, given the severity of admitted patients, hospitalization in AN offers unique challenges for both patients and clinicians, mostly in the context of a very acute and not infrequently unplanned admission (i.e., through the emergency room). If the latter is the case, patients do not undergo the preliminary steps in treatment that could promote their motivation; therefore, they find themselves accepting a high-intensity therapeutic condition without being engaged in treatment and aware of needing it (7).

Although it is an everyday clinical experience that readmissions (RAs) in AN are fairly common, currently, this kind of outcome has received scant attention. In fact, in spite of providing patients with specific discharge plans (6), a substantial number of patients require multiple hospitalizations, sometimes over a short period of time. There are few longitudinal studies in AN on inpatients, and RA is rarely considered as an outcome itself, with most works focusing instead on the stabilization/improvement of outcome measures at follow-up (8–11). Earlier data suggested RAs as becoming increasingly frequent over time (12), but no other data are currently available for adults with AN. In contrast, literature on adolescents with AN paid closer attention to rehospitalizations, with studies supporting previous RAs, young age, low socioeconomic status, co-occurring illnesses, and poor rate of weight gain during hospitalization as predictors of rehospitalization (13–15). However, rehospitalizations in adolescents with AN seem to be quite uncommon (16), and the available data may not apply to adults.

The “revolving door” (RD) phenomenon defines those patients who undergo multiple hospitalizations in a relatively short time. In the field of psychiatry, since its first description for alcoholism (17), the analysis of RD has been mostly applied to affective and non-affective psychoses. For example, research showed that RD patients with bipolar disorders had more frequently mixed episodes or medical comorbidities (18) than non-RD individuals. Similarly, patients with schizophrenia reported more often RD in the case of greater severity of psychotic symptoms, lifetime substance use, and premature discharge (19) compared with non-RD individuals. Despite its utilization, the RD definition remains polyform, potentially echoing the specificities of the samples taken into account. Consequently, the definition of RD ranges from two (5, 20, 21) or three or more hospitalizations in the last year (18) to three or more psychiatric admissions in 2 years (22) to three hospitalizations during lifetime (23).

Given the aforementioned gaps in the literature, with this study, we aimed to expand current knowledge on the characteristics of patients who require RA in AN (RA-AN) and the frequency and predictors of rehospitalization. Consistently with earlier research, we defined RD patients with AN (RD-AN) as those who required RA within 12 months since last discharge. With more detail, adopting a longitudinal design, we aimed to (a) identify and characterize patients with AN who needed to be readmitted (i.e., RA-AN) and those who required such an early hospital RA to meet criteria for RD (RD-AN), (b) ascertain eventual differences between patients with RA-AN and those without reutilization of the hospital stay (i.e., RA-AN vs. non-RA-AN) during their baseline hospitalization, (c) investigate as to whether clinical or psychometric parameters worsened on RA, and (d) analyze time to RA in RA-AN and ascertain the predictors of rehospitalization, focusing on clinical variables of patients at baseline admission (i.e., diagnostic subtype, eating, general psychopathology, and clinical variables) and their outcome at baseline hospitalization (i.e., weight improvement).

We expected to determine a substantial number of patients being RA-AN and meeting the RD-AN criteria since it is a common clinical experience that a relevant proportion of those who need RA do so over the short run. We also *a priori* hypothesized that patients with RA-AN would respond more poorly to baseline hospitalization than the non-RA-AN counterpart and that RA-AN would show poorer BMI on RA compared with baseline hospitalization. Concerning the predictors of time to RA, we hypothesized greater clinical severity (i.e., low BMI, severe eating psychopathology, and anxiety and depressive symptoms) and poorer response to baseline hospitalization (i.e., poor BMI increase during hospitalization) as the potential predictors of RA in AN.

## Materials and Methods

### Participants

A total of 186 patients with AN, voluntarily admitted to the hospitalization program at the Eating Disorders Center of the “Città della Salute e della Scienza” hospital at the University of Turin, Italy, were consecutively enrolled from March 2013 to December 2017. The recruitment for this study was terminated to perform at least 3 years of longitudinal observation (i.e., until December 2020) for all participants. Consequently, we could identify and report also eventual RAs during 36 months after discharge. We labeled patients with AN who needed to be readmitted after their baseline hospitalization discharge as RA-AN and those who required RA within 12 months since last discharge as RD-AN.

The inclusion criteria were as follows: (a) diagnosis of AN as assessed by an experienced psychiatrist with the Structured Clinical Interview for Diagnostic and Statistical Manual of Mental Disorders, fifth edition (DSM-5) (24), (b) age >16 years old, and (c) no psychotic or bipolar disorders. Of all candidates, 12 returned incomplete assessments, and 4 refused study participation. Finally, 170 inpatients with AN were included in this study for which written informed consent was provided by all patients (or parents in the case of age of patient <18 years old). This study was approved by the Ethical Committee of the “Città della Salute e della Scienza” hospital at the University of Turin, Italy, with protocol number 0036472.

### Treatment

All participants were inpatients treated at the specialist eating disorder (ED) unit of the Eating Disorders Center of the “Città della Salute e della Scienza” hospital, University of Turin, Italy. The majority of patients (over 80%) were admitted through the emergency room in a very acute phase of AN. The international guidelines underlie treatment delivery so the clinical team is multidisciplinary (psychiatrists, clinical psychologists, psychiatric nurses, internal medicine physicians, and registered dietitians) and extensively experienced. In addition, treatment is delivered following the requirements and specificities needed when dealing with patients with AN who need inpatient treatment (25, 26). Since several patients are hospitalized because of an emergency condition and without a pre-hospitalization treatment plan fostering motivation, the intervention is focused on the following aims: to re-establish patients' clinical life-threatening conditions, work with the patients (twice per week) to foster their motivation for the subsequent therapeutic steps, deliver structured daily sessions on symptom management focusing on diet and body image concerns, work psychologically (twice per week) to understand the possible causes of those factors that led to an emergency admission, and provide families with psychoeducation. Individualized treatment plans are provided (27), and behavioral contracting about meals and eating symptoms is performed. Parenteral/enteral nutrition is proposed according to individual needs; given the severity of the patients, the nasogastric tube can also be required to avoid refeeding syndrome.

### Materials

Sociodemographic and clinical characteristics of the patients were collected upon hospital admission (T0) and discharge (e.g., end of treatment, EOT) with a clinical interview during each hospitalization event (i.e., H1: baseline admission; H2: readmission). Body mass index (BMI) was obtained by clinicians after measurement of height and weight of the patients at both time points for each hospitalization.

All participants completed the following assessments at both T0 and EOT:

- Eating Disorder Inventory-2 (EDI-2), Italian validation (28): the questionnaire evaluates the eating-related pathology. The present study considered the first three symptomatic subscales of the tool, namely, drive for thinness (DT), bulimia (B), and body dissatisfaction (BD), as they assess the attitudes toward eating, weight, and body (29). Higher scores in each subscale suggest a greater severity of the measured symptom. The Italian version of the questionnaire has a good internal consistency [Cronbach alpha value >0.90 (30)].
- State-Trait Anxiety Inventory [STAI (31)]: two sets of 20 questions measure the state anxiety (i.e., the current level of anxiety) and the trait anxiety (i.e., anxiety as a stable trait). Participants range on a scale from 1 (never) to 4 (always). The internal consistency is good with alpha Cronbach values between 0.86 and 0.95 (32).
- Beck Depression Inventory [BDI; (33)]: the 13-item questionnaire assesses depressive symptoms severity as follows: a global score between 0 and 4 corresponds to low/minimal symptoms, scores from 5 to 15 indicate mild/moderate depression, while rates from 16 to 39 reveal severe depressive symptomatology. The internal consistency is good, with an alpha Cronbach value of 0.86 (34).
- Body Shape Questionnaire [BSQ (35)]: this tool evaluates body image and body dissatisfaction. It consists of 34 items asking for feelings of the patients on body shape during the last weeks. Higher scores indicate higher levels of body dissatisfaction. The internal consistency is good, with Cronbach's alpha values between 0.82 and 0.89 (36).

In case of re-hospitalization, patients completed, in addition to STAI and BDI, the following:

- Eating Disorder Examination Questionnaire (EDE-Q), Italian version (30): the questionnaire measures the occurrence of typical behaviors of eating disorders during the last 28 days. It provides four subscales (dietary restraint, eating concerns, weight concerns, and shape concerns) and a global score. Higher scores correspond to higher eating-related psychopathology. The internal consistency of the Italian version is good with a Cronbach alpha value of 0.90 (30).

### Statistical Analysis

To compute the analysis, the SPSS 27.0 statistical software package (IBM SPSS Statistics for Windows, Version 27.0., IBM Corp, Armonk, NY, USA) was used.

A paired-sample *t*-test was used to calculate differences between patients' baseline characteristics between baseline (T0 at H1) and second (T0 at H2) hospitalization. Independent sample *t*-test and Fisher's exact test were used to compare groups reporting RA (RA-AN) or not (non-RA-AN) for continuous and categorical variables, respectively. Repeated measure ANOVA was run to verify the eventual differences in clinical outcome between RA-AN and non-RA-AN during the first hospitalization. The difference in BMI between T0 at H1 and EOT at H1 was calculated as ΔBMI.

Percentiles were used to categorize the variables of interest as measured at the beginning of the baseline hospitalization (i.e., T0 at H1). For example, patients were categorized as follows: high DT in case of score >15 (50th percentile of the whole sample). To assess clinical outcome during H1, the 50th percentile of ΔBMI was calculated. Log-rank-tests were run to compare time-to-readmission survival curves between those with high vs. low scores on those measures that significantly differed between RA-AN and non-RA-AN groups (i.e., age, duration of illness, BMI, DT, B, BD on the EDI-2, BDI, STAI-S, BSQ, and ΔBMI). Subsequently, Cox regressions (proportional hazard regressions) were used in order to clarify whether a certain baseline variable (i.e., high vs. low DT) could be significantly associated with time-to-readmission also after statistical control for confounders, namely, those variables that differed between RA-AN and non-RA-AN groups.

## Results

### Sociodemographic and Clinical Characteristics of the Sample

Patients were all Caucasian and voluntarily admitted. None of them left the program against medical advice. Of the 170 patients, 124 (72.9%) were diagnosed with the restricting type of AN (AN-R), while 46 (27.1%) were diagnosed with the binge-purging (AN-BP) subtype. The mean age of the sample was 24.8 ± 9.6 years, the mean duration of illness was 6.5 ± 8.2 years, and the mean BMI was 14.2 ± 1.7 (i.e., overall extreme AN according to the DSM-5 severity specifiers). The mean duration of H1 was 35.5 ± 16.7 days, and that of H2, for those requiring it, was 29.1 ± 14.6 days.

### Readmission and Revolving Door in Anorexia Nervosa

**Figure 1:**
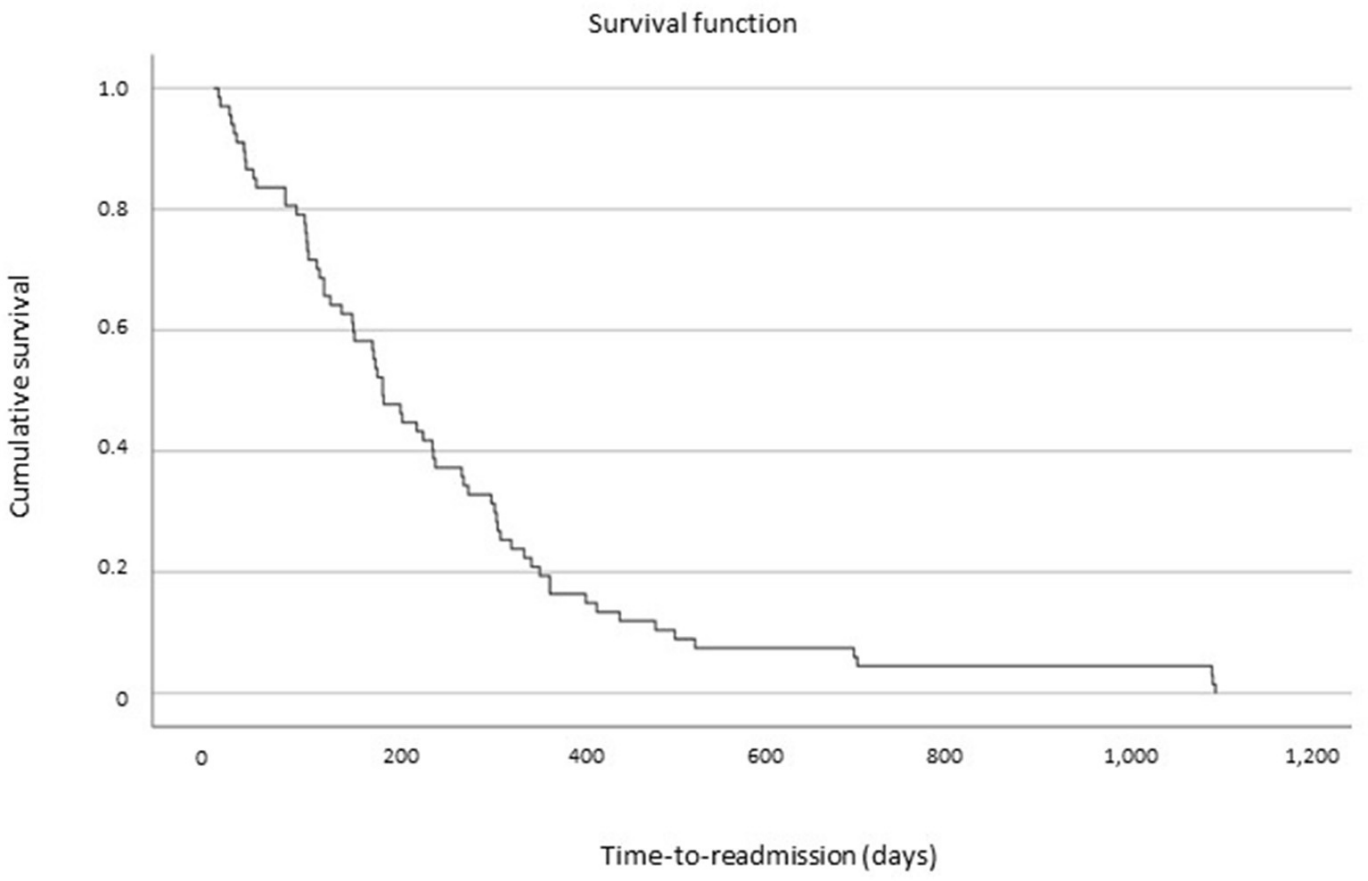
Survival analysis after baseline discharge of time-to-readmission for patients with anorexia nervosa.

A total of 67 (RA-AN; 39.4%) patients required to be rehospitalized after H1, and 62 of them (92.5% of RA-AN) met the criteria for being classified as RD patients (i.e., at least an RA within 12 months since last discharge; RD-AN). In the RA-AN group (*n* = 67), 34 patients needed only one RA after H1 discharge (20% of the whole sample), while 33 (19.4% of the whole sample) required more than two admissions after H1 discharge (range, 3–15 hospitalizations). All RAs were at our specialist ED inpatient unit. The survival analysis is shown in Figure 1.

### Differences in Clinical Variables and Outcome Between RA and Non-RA Patients With Anorexia Nervosa at Their Baseline Hospitalization

No differences emerged between RA-AN and non-RA-AN concerning gender (Fisher's exact test, *p* = 1), whereas patients with AN-BP were classified as RA-AN more frequently than those with AN-R (RA-AN: AN-BP, 37.3%; AN-R, 62.7%; and non-RA-AN: AN-BP, 20.4%; AN-R, 79.6%; Fisher's exact test, *p* = 0.021). As shown in Table 1, non-RA-AN and RA-AN did not differ on the number of previous hospitalizations. In addition, non-RA-AN and RA-AN differed concerning age, duration of illness, drive for thinness (DT), bulimia, and body dissatisfaction (BD) subscales on the EDI-2; depressive symptoms; state anxiety; and body image concerns as measured by BDI, STAI-S, and BSQ, respectively. No differences emerged concerning psychiatric comorbidity as well or major depression (RD-AN, 36.5%; non-RD-AN, 35%; Fisher's exact test, *p* = 0.868) or anxiety disorders (RD-AN, 9.5%; non-RD-AN, 11%; Fisher's exact test, *p* = 1).

**Table 1:** Differences in clinical variables during baseline hospitalization between readmitted (RA-AN) and non-readmitted (non-RA-AN) patients with anorexia nervosa (AN).

	**Patients with AN** ***n*** **= 170**			
	**Non-RA-AN** ***N* = 103**	**RA-AN** ***N* = 67**	**Test statistics**	
	**Mean (SD)**	**Mean (SD)**	***t***	***p***
Age, years	26.1 (10.6)	22.9 (7.6)	2.1	**0.035**
Duration of illness, years	7.5 (9.3)	4.9 (5.8)	2	**0.042**
Number of previous hospitalizations	1 (2)	1.3 (2.6)	0.71	0.478
Body mass index	14.1 (1.7)	14.4 (1.8)	1.03	0.305
Duration of first hospitalization, days	35.7 (18.8)	35.2 (12.8)	0.2	0.841
EDI-2				
Drive for thinness	10.9 (7.8)	14.7 (7.2)	2.97	**0.003**
Bulimia	2.4 (4)	4.4 (5.4)	2.67	**0.008**
Body dissatisfaction	13.3 (6.7)	16 (7)	2.35	**0.020**
BDI	14.4 (7.7)	18.5 (7.8)	3.13	**0.002**
STAI-T	55.3 (13.2)	59.3 (14.5)	1.75	0.082
STAI-S	52.4 (14.3)	57.3 (14.3)	2.03	**0.044**
BSQ	114.2 (45.9)	132.5 (42.2)	2.54	**0.012**

*EDI-2, Eating Disorders Inventory-2; BDI, Beck Depression Inventory; STAI-T, State-Trait Anxiety Inventory-Trait; STAI-S, State-Trait Anxiety Inventory-State; BSQ, Body Shape Questionnaire. Bold values mean p < 0.05*.

Concerning the outcome, both groups significantly improved on all considered outcomes between T0 and T1 at H1 (i.e., BMI, EDE-Q total score, BDI, STAI-S, BSQ; see Supplementary Material) with no significant interactions between the considered variables and RA-AN/non-RA-AN groups. After H1 discharge, no differences emerged concerning post-discharge treatment plans (RA-AN, outpatient service, 49.2%; day hospital/residential, 50.8%; and non-RA-AN, outpatient service, 47.5%; day hospital/residential, 52.5%; Fisher's exact test, *p* = 0.871). After discharge from baseline hospitalization, all patients received outpatient treatment.

### Differences Between Baseline Hospitalization and Readmission for RA-AN

When analyzing the changes in the scores of the patients over time comparing subsequential RAs (i.e., H1 and H2), the BMI improved, while the BDI significantly worsened in the same timeframe (see Table 2). Both STAI-S and EDE-Q total score did not show any changes when H1 and H2 scores were compared.

**Table 2:** Differences across baseline and second hospitalization for patients with AN reporting (RA-AN) or not (non-RA-AN) readmissions after baseline discharge.

	**RA-AN** ***N*** **= 67**			
	**T0 at H1**	**T0 at H2**	**Test statistics**	
	**Mean (SD)**	**Mean (SD)**	***t***	***p***
BMI	14.5 (1.7)	15.5 (3.2)	2.92	**0.005**
BDI	18.8 (7.7)	21.3 (7.9)	2.59	**0.013**
STAI-S	58.1 (14.5)	61.6 (13.8)	1.78	0.081
EDE-Q total score	4 (1.5)	4 (1.6)	0.014	0.989

*T0, hospital admission; H1, baseline hospitalization; H2, readmission; BMI, body mass index; BDI, Beck Depression Inventory; STAI-S, State-Trait Anxiety Inventory-State; EDE-Q, Eating Disorder Examination Questionnaire. Bold values mean p < 0.05*.

### Survival Analysis of Time to Readmission in Anorexia Nervosa

The 13.4% of patients classified as RA-AN required RA within the first 30 days since discharge, while the mean time to RA was 251.8 ± 239.6 days. Patients with AN-R did not differ from those with AN-BP in terms of time to RA (AN-R mean days, 274.6; AN-BP mean days, 213.7; log-rank test, *p* = 0.23) as well as patients with an enduring duration of illness [set at 7 years per earlier literature (37), ≥7 years of AN mean days, 246.7; <7 years of AN mean days, 257.4; log-rank test, *p* = 0.604].

After splitting the sample according to the 50th percentile of the variables that differed between groups (i.e., high vs. low age, duration of illness, BMI, and DT, bulimia, and BD on the EDI-2, BDI, STAI-S, and BSQ), a different time to RA emerged for patients with short vs. long duration of illness (short duration mean days, 190.3; long duration mean days, 308.9; log-rank test, *p* = 0.042) and low vs. high DT at first hospitalization (low DT mean days, 334.5; high DT mean days, 203.1; log-rank test, *p* = 0.025). In contrast, no significantly different survival times emerged comparing patients with high vs. low age (younger age mean days, 222.2; older age mean days, 286.3; log-rank test, *p* = 0.399), bulimia (low bulimia mean days, 306.7; high bulimia mean days, 216.4; log-rank test, *p* = 0.222), and BD (low BD mean days, 265.4; high BD mean days, 240.4; log-rank test, *p* = 0.613) subscales on the EDI-2, BMI (low BMI mean days, 308; high BMI mean days, 212.8; log-rank test, *p* = 0.124), BDI (low BDI mean days, 235.3; high BDI mean days, 260.1; log-rank test, *p* = 0.877), STAI-S (low-STAI-S mean days, 259.3; high-STAI-S mean days, 244.2; log-rank test, *p* = 0.442), and BSQ (low BSQ mean days, 314.8; high BSQ mean days, 223.4; log-rank test, *p* = 0.124) measured upon baseline hospital admission of the patients.

With respect to the outcome of baseline hospitalization, we considered in the analysis the weight gain of the patients during hospitalization (ΔBMI), splitting the sample according to the 50th percentile (i.e., ΔBMI = 0.7). Patients with low vs. high weight gain during hospitalization significantly differed in time to RA (low-ΔBMI mean days, 208.7; high-ΔBMI mean days, 351.4; log-rank test, *p* = 0.017).

### Predictors of Time to Readmission in Anorexia Nervosa

As shown in Table 3, when examining the predictors of shorter time to RA, patients with shorter duration of illness reported an increased likelihood of early RA compared with those with a longer duration of illness. However, this finding did not survive statistical control for confounders. Instead, as shown in Figure 2, patients with high DT were significantly associated with a greater risk of being readmitted earlier than those with low DT (see also Table 3). This result held significance even after statistical control for age, duration of illness, EDI-2 subscales bulimia and BD, STAI-S, BSQ, and BDI and ΔBMI, namely, those variables that significantly differed between the RA-AN and non-RA-AN groups at baseline hospitalization. Similarly, patients with low ΔBMI were significantly associated with a greater risk of being readmitted earlier than those with high ΔBMI (see Table 3). As for DT, this result held significance after statistical control for confounders (i.e., age, duration of illness, EDI-2 subscales bulimia and BD, STAI-S, BSQ, and BDI). However, when baseline DT was also added to the model, its significance was lost. High vs. low age, BMI, bulimia and BD (EDI-2), BDI, STAI-S, and BSQ did not result as significant predictors of time to RA in AN.

**Table 3:** Predictors of time-to-readmission in anorexia nervosa.

	**Uncorrected model**			**Model 1**			**Model 2**		
	**Wald's test**	***p***	**Hazard ratio (95% CI)**	**Wald's test**	***p***	**Hazard ratio (95% CI)**	**Wald's test**	***p***	**Hazard ratio (95% CI)**
**H1 baseline variables**									
Young age vs. old age	0.7	0.4	1.2 (0.7–2)						
Long vs. short duration of illness	4	**0.045**	0.6 (0.4–0.9)	1.9	0.167	0.55 (0.2–1.3)			
High DT vs. low DT	4.8	**0.028**	1.9 (1.1–3.4)	4.2	**0.043**	4.2 (1.1–16.4)	4.1^*^	0.045^*^	4.4^*^ (1.1–18.5)
High B vs. low B	1.5	0.226	1.4 (0.8–2.5)						
High DT vs. low DT	0.25	0.615	1.1 (0.7–1.9)						
Low BMI vs. high BMI	2.3	0.13	0.67 (0.4–1.1)						
High BDI vs. low BDI	0.24	0.87	1 (0.6–1.9)						
High STAI-S vs. low STAI-S	0.58	0.44	1.2 (0.7–2)						
High BSQ vs. low BSQ	2.3	0.13	1.6 (0.9–2.8)						
**H1 outcome variable**									
Low ΔBMI vs. high ΔBMI	5.6	**0.018**	1.9(1.1–3.3)	5.3	**0.021**	2.3 (1.1–4.4)	3.8^#^	0.5^#^	2^#^(0.9–4)

*H1, baseline hospitalization; DT, Drive for thinness; BMI, Body Mass Index; BDI, Beck depression Inventory; STAI-S, State-Trait Anxiety Inventory-State; BSQ, Body Shape Questionnaire*.

*Model 1: Corrected model for age, duration of illness, EDI-2 subscales bulimia and body dissatisfaction, STAI-S, BSQ, and BDI*.

*Model 2: Corrected model for age, duration of illness, EDI-2 subscales bulimia and body dissatisfaction, STAI-S, BSQ, and BDI, and DT or ΔBMI*.

* *Model 2 corrected also for ΔBMI*.

# *Model 2 corrected also for DT. Bold values mean p < 0.05*.

**Figure 2:**
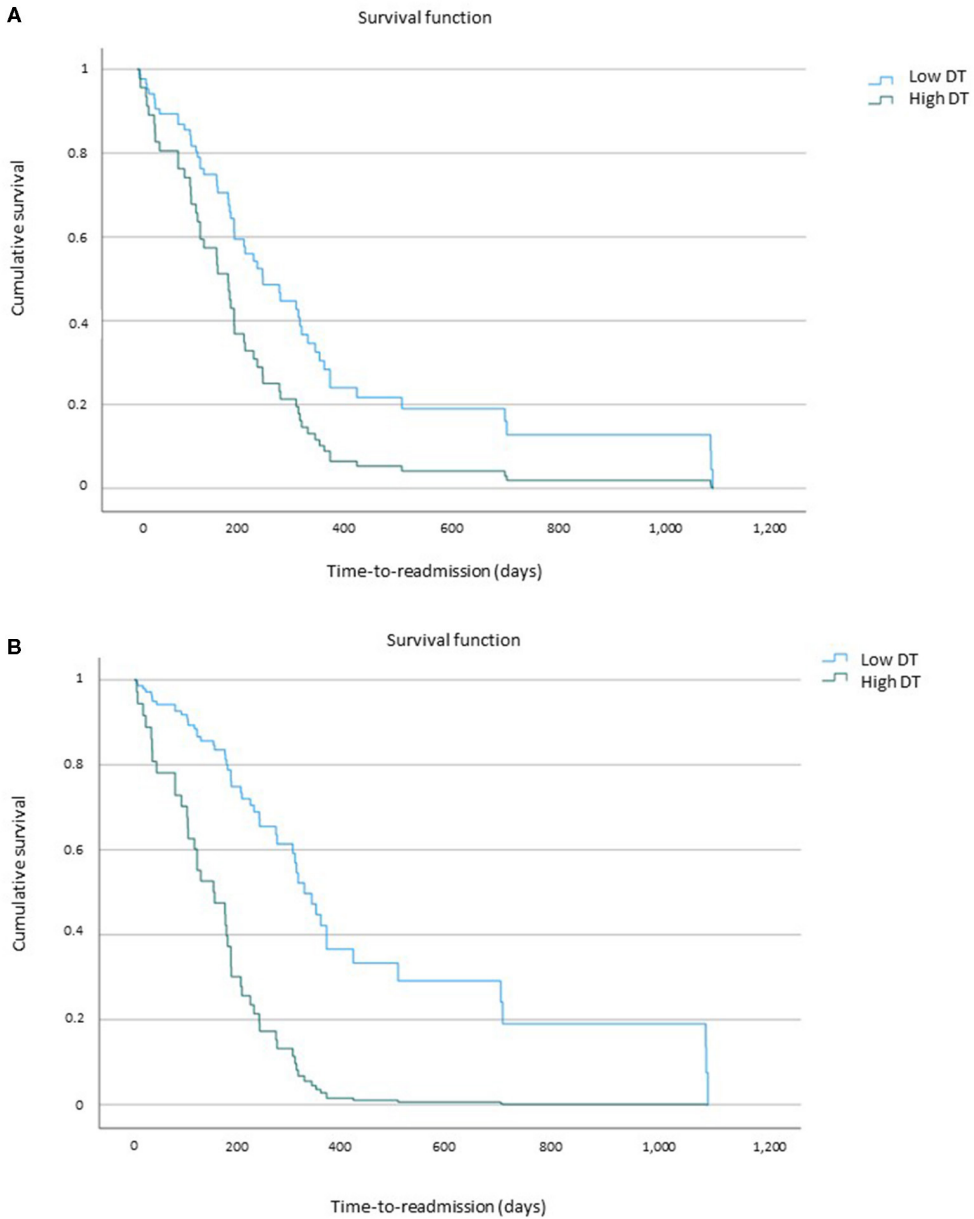
High drive for thinness (DT) as a predictor of shorter time-to-readmission. **(A)** Uncorrected model of high drive for thinness (DT) as a predictor of time-to-readmission. **(B)** Corrected model (statistical control for age, duration of illness, EDI–2 bulimia and body dissatisfaction, STAl-S, BDl, and BSQ) of high drive for thinness as a predictor of time-to-readmission.

## Discussion

With this study, we aimed to investigate the patterns of rehospitalization in inpatients with severe AN measuring readmissions, frequent readmissions, and predictors of time-to-readmission. Four main findings emerged: first, a substantial number of patients (40%) required readmission (RA-AN), and the vast majority of the latter group (62 of 67 patients) met the criteria for RD (i.e., rehospitalization in the first 12 months after discharge). Second, the RA-AN and non-RA-AN groups responded equally well to their baseline hospitalization; third, when needing RA, the BMI of the patients improved, while depressive symptoms worsened. Finally, a shorter duration of illness predicted early RA but did not hold significance after statistical control for confounders. In contrast, high baseline levels of DT significantly predicted early RA independently of all baseline differences between groups as well as BMI improvement during the previous hospitalization. In addition, the improvement of BMI during baseline hospitalization was found to be a robust predictor of time to RA, but when DT was added to the model, it did not hold significance.

Overall, these are novel findings in the field of AN, since no longitudinal data were currently available on time trends before rehospitalization and predictors of time to RA in adult patients with AN. In line with the *a priori* hypothesis, our results suggest an RA rate (67 of 170 patients, 39.4%) in line with an earlier research (38), but this finding also helps expand knowledge on the time required to be readmitted since the vast majority (92.5%) of patients with AN who needed RA did so within 12 months since their last discharge. On the one hand, this finding highlights the need for a fluid transition between inpatient and outpatient services, as already advocated (6), also in other fields of psychiatry (39). The transition between inpatient and outpatient services has long been considered a possible contributing cause of frequent RAs with estimates of a lack of a link to outpatient care after an acute hospitalization ranging from 22 to 90% (40). In this vein, interventions that bridge the transition home, thus, increasing community support, have been recently authoritatively advocated for AN (41). On the other hand, it should also be noted that individualized discharge plans could have helped avoid even more rapid patterns of RAs; in fact, different from earlier studies on general psychiatry (42), the first month since hospital discharge was not a critical period for RA. Notably, the RA-AN and non-RA-AN groups did not show differences in post-discharge plans, and partial hospitalization was delivered to a substantial number of cases to minimize the inpatient–outpatient dichotomy, in keeping with guidelines (6) and literature suggesting day hospital interventions as a significant tool of continuing care after hospitalization (43). Finally, our findings are also in line with an earlier research on predictors of relapse (44) reporting that 41% of participants relapse during the 1-year follow-up period and that the highest risk of relapse occurs between 4 and 9 months post-treatment.

Of note, when analyzing the differences between RA-AN and non-RA-AN at the entry of their baseline hospitalization, several differences emerged. First, patients in the RA-AN group were younger and had a lower duration of illness. This is of interest, since this finding may indicate the need for specific therapeutic plans for the youngest patients, who instead tend to show duration of untreated illness even longer than the adults (45). In addition, this datum is in line with earlier studies on poor reliability of duration of illness as a proxy for clinical severity of AN (46–48). Patients with RA-AN were more frequently diagnosed with BP-AN (and, relatedly, reported a higher bulimia score on the EDI-2), in line with data showing patients with BP-AN as poor responders (49) and being more susceptible to relapse after treatment (44). Although the groups did not differ in psychiatric comorbidity, thus, supporting an intertwined relationship between AN and anxiety and depression (50), such symptomatologies were more pronounced in the RA-AN group than in the non-RA-AN group. Patients with RA-AN reported a trend of greater severity also concerning body image; notwithstanding, the BMI did not differ between groups, in line with literature questioning the utility of BMI itself as a severity specifier (51).

When evaluating the outcome of baseline hospitalization, both groups showed a significant improvement on all measures considered, different from what we hypothesized. Therefore, putting this datum in perspective, no differences in response to baseline hospitalization emerged between those who, months later, would have required RA or not. In addition, the mean length of baseline hospitalization was comparable between groups and in line with earlier data (52). However, it is noteworthy that no early discharge emerged for RA-AN, since both groups remained hospitalized for a similar time; again, no consensus exists on the role of early discharge and frequent RAs, with the available literature on other mental disorders both supporting (19) and opposing (22) difference in length of stay between RD and non-RD patients. As already outlined earlier, no differences emerged in discharge plans, different from data about bipolar disorders (18).

Different than expected, it is of note that, comparing BMI at the entry of baseline hospitalization vs. RA, the RA-AN group reported improved BMI, thus, suggesting a different main cause requiring hospitalization. Echoing this finding, also eating psychopathology and anxiety symptoms were overall stable, while depressive symptoms significantly worsened. This is of interest since it has been suggested that the causes for RA could include significant weight loss (15), while our data suggest that this is not the main factor since depressive symptoms seem to take the lion's share in this regard. Earlier research suggested that comorbidity is substantially independent of BMI (53) and that depressive aspects can become even more prominent after the improvement of ED (54). Finally, it has been reported that depressive symptoms are correlated with the course of body image disturbances; in fact, during treatment and the related BMI improvement, the correlation of symptoms of depression and body image perceptions increased (55). Therefore, this could be a relevant factor influencing the course of AN, and further research is needed to investigate depressive symptoms as maintaining factors in AN.

Echoing the young age of patients in the RA-AN group, a shorter duration of illness (<3 years) predicted early RA, in keeping with literature questioning duration of AN as a reliable severity specifier (46–48). However, this predictor did not hold significance after statistical control, so other factors (i.e., depressive symptoms) could have influenced this finding. Interestingly, high levels of DT robustly predicted a shorter time before RA for patients with severe AN. In contrast with the *a priori* hypothesis on other potential predictors, this datum is in line with what we hypothesized when designing this study. This finding on DT as a predictor of time to RA expands knowledge on the longitudinal outcome of AN since time to RA had not been investigated so far. With more details, the available studies on longitudinal outcomes in AN focused on good vs. poor clinical outcomes (9), potentially underreporting data on RA itself, which could be generically labeled as poor outcome. We focused on DT because it is a core component of AN psychopathology (29). It has been recently proposed as a reliable severity specifier for AN (56), and earlier research found DT to be associated with poor outcome (9). Interestingly, DT predicted early RA even against statistical control (actually, gaining relevance after statistical control) of all clinical (age, duration of illness, body image concerns, and improvement in BMI) and comorbidity-related (depressive and anxious symptoms) aspects, notwithstanding their relevance in AN (55–57). In keeping with data on positive outcomes at follow-up in the case of high BMI at discharge (10), our findings showed that improvement in BMI during baseline admission predicted time to RA, also after statistical control. This is of importance since our sample was composed of individuals with extreme AN (BMI <15) facing an acute phase of the disorder. Such a difficult condition is particularly challenging in the light of data on unfavorable outcomes for those who require emergency hospitalizations (58) and for whom no pharmacological agents showed effectiveness (59). In fact, these patients experience a very acute phase of AN without receiving the recommended motivational preparation and engagement performed when hospitalizations are planned instead of required because of a life-threatening emergency. In addition, certain BMIs require a cautious approach (27), and weight gain is particularly complex to achieve. However, the significance of improvement in BMI did not hold significance when DT was added to the model as a confounder (while the opposite, i.e., DT controlled for improvement in BMI, survived statistical control). Therefore, our data support earlier studies suggesting that “it should not be expected that weight gain alone will ultimately confer commensurate psychological symptom remission” [(3), p. 542]. According to our findings, the hypothesis can be raised that the increase in BMI, if coupled with high DT, does not exert a protective effect on RAs. Therefore, clinicians should pay closer attention to the improvement of both aspects, mostly for patients with heightened DT. Earlier data from our group showed that DT during hospitalization can even worsen, despite the improvement of all other clinical parameters (60).

In conclusion, our study provided evidence on a frequently overseen aspect of AN, namely, the time trends before rehospitalization and related predictors. Significantly, patients with AN showed a pattern of frequent utilization of the inpatient facility, although the RA-AN and non-RA-AN groups responded equally well to baseline hospitalization. In addition, BMI and eating psychopathology improved, and depressive symptoms worsened when RA became necessary. The subtype of AN, psychiatric comorbidity, duration of illness, and depressive and anxious symptoms did not predict early RA, while DT was found to be the strongest predictor of time to RA for patients with severe AN, independent of several other well-known components of the clinical constellation of symptoms in AN, including anxiety, depression, and body image concerns. Moreover, improvement in BMI during hospitalization was found to be the other key predictor of the amount of time required before RA, although to a smaller extent than high scores of DT. As such, future research is needed to confirm these findings and overall promote the investigation of factors potentially involved in the clinical trajectory of patients in such an acute condition of AN. In fact, in the era of precision medicine, individualized treatment approaches should be proposed to patients who suffer from such a pernicious condition like (extreme) AN.

In spite of several strengths including the sizable sample, longitudinal design, real-world context, and specialized treatment delivered, this study has some limitations: the severity of included patients with AN and the data collection at an academic specialized ED unit could hamper data generalizability. Moreover, the sample size was not large enough to perform a comparison between RA-AN and RD-AN, and all patients reported prior hospitalizations, although not differing between groups. Notwithstanding, these data could have interesting treatment implications. In line with recent data on the cognitive–affective biases about one's own body in AN (61), baseline DT upon admission, and BMI improvement while hospitalized should be both taken into account when designing individualized treatments and post-discharge plans for patients with AN, even for those who are acutely ill.

## Data Availability Statement

Data available on request due to privacy/ethical restrictions.

## Ethics Statement

This study was approved by the Ethical Committee of Città della Salute e della Scienza hospital at the University of Turin, Italy with protocol number 0036472. Written informed consent to participate in this study was provided by all participants and by the participants' legal guardian/next of kin, when needed.

## Author Contributions

EM and GA-D conceived and designed the study. ND, PL, and FS conducted the assessments. EM and PL drafted the manuscript and ran the analyses. FS and GA-D supervised the analyses. ND and GA-D critically revised the manuscript. All authors approved the final article.

## Conflict of Interest

The authors declare that the research was conducted in the absence of any commercial or financial relationships that could be construed as a potential conflict of interest.

## Publisher's Note

All claims expressed in this article are solely those of the authors and do not necessarily represent those of their affiliated organizations, or those of the publisher, the editors and the reviewers. Any product that may be evaluated in this article, or claim that may be made by its manufacturer, is not guaranteed or endorsed by the publisher.
