# Supplementary material for: Rehospitalization and “Revolving Door” in Anorexia Nervosa: Are There Any Predictors of Time to Readmission?
Source: Front Psychiatry. 2021 Jul 23;12:694223. doi: 10.3389/fpsyt.2021.694223 (PMC8342847; doi:10.3389/fpsyt.2021.694223)
Supplement: Supplementary file 1 [file Table_1.DOCX]

***Table S1. Clinical changes during baseline hospitalization between readmitted (RA-AN) and non-readmitted (non-RA-AN) patients with AN.***

|  | Non-RA-AN  n=103 | | RA-AN  n=67 | | Test statistics | | | | | | | | |
| --- | --- | --- | --- | --- | --- | --- | --- | --- | --- | --- | --- | --- | --- |
|  | H1 T0 | H1 EOT | H1 T0 | H1 EOT | Main effect of time | | | Main effect of time*group | | | Main effect of group | | |
|  | Mean(SD) | Mean(SD) | Mean(SD) | Mean(SD) | F | p | η^2^ | F | p | η^2^ | F | p | η^2^ |
| BMI | 14.1(1.7) | 15.1(1.7) | 14.4(1.8) | 15(1.5) | 83.7 | **<.001** | .348 | 2.6 | .110 | .016 | .2 | .696 | .001 |
| EDE-Q total score | 3.2(1.7) | 2.3(1.7) | 3.8(1.6) | 3.0(1.5) | 58.2 | **<.001** | .318 | .01 | .931 | .000 | 5.2 | **.024** | .040 |
| BDI | 13.7(7.4) | 9.7(7.9) | 17.8(8.3) | 12.9(8.4) | 53.1 | **<.001** | .303 | .6 | .450 | .005 | 7.1 | **.009** | .055 |
| STAI-S | 52.2(14.3) | 46.8(15.8) | 58.8(14.5) | 55.9(14.6) | 12.3 | **.001** | .092 | 1.1 | .286 | .009 | 9.5 | **.003** | .073 |

Legend: H1: baseline hospitalization; T0: hospital admission; EOT: end of treatment; BMI = Body Mass Index; EDE-Q = Eating Disorders Examination Questionnaire; BDI = Beck Depression Inventory; STAI-S = State-Trait Anxiety Inventory – State.
